# Usage of Tranexamic Acid for Treatment of Subdural Hematomas

**DOI:** 10.7759/cureus.37628

**Published:** 2023-04-15

**Authors:** Michael Wu, Hassaan Wajeeh, Marissa N McPhail, Omar Seyam, Jamie Flora, Hoang Nguyen

**Affiliations:** 1 Osteopathic Medicine, Nova Southeastern University Dr. Kiran C. Patel College of Osteopathic Medicine, Fort Lauderdale, USA; 2 Basic Sciences, Nova Southeastern University Dr. Kiran C. Patel College of Osteopathic Medicine, Clearwater, USA

**Keywords:** middle meningeal artery embolization, open craniotomy, tranexamic acid, subdural hematoma (sdh), chronic subdural hematoma (csdh)

## Abstract

The collection of blood in the subdural layer within the cranium is classified as a subdural hematoma. Prevalence of subdural hematomas is most common among older populations with the current standard of treatment being invasive surgical evacuation for patients presenting with acute subdural hematomas with a midline shift greater than 5 mm on computed tomography (CT).Tranexamic acid (TXA) has been identified as an alternative, non-invasive option to treat patients presenting with subdural hematoma who are not suitable for surgical intervention.

The presenting case involves a 90-year-old female who arrived with a code stroke with the chief complaint of right lower extremity weakness. A stroke series CT panel revealed a left frontal multiloculated subdural hematoma, measuring 130 mL with mass effect and a midline shift of 7 mm. The patient was recommended a craniotomy for hematoma evacuation or access to hospice for comfort care. A second opinion resulted in the administration of TXA. After the full completion of a TXA course, the patient achieved baseline mobility. The final measurements revealed a final hematoma volume of 10 mL and a midline shift of less than 2 mm.

Current literature, as well as the case described, has begun demonstrating the efficacy of the usage of TXA in the reabsorption of subdural hematomas and should encourage further exploration into society guidelines for the usage of TXA as a non-invasive alternative to treat subdural hematomas.

## Introduction

Subdural hematoma is an injury common amongst the elderly after traumatic events and displays a prevalence of 1.7 - 20.6 per 100,000 people [1]. A subdural hematoma develops after tearing the bridging veins within the dura, subsequently leading to the collection of blood between the arachnoid and dura layers [2]. The dura is the tough outer layer of tissue protecting and encapsulating the brain. In the pathological state, the dura is responsible for the formation of subdural hematomas by trapping the blood leaking from torn bridging veins [3]. Subdural hematomas can be classified as either acute, subacute, or chronic. Subacute hematomas occur within four to 21 days of a head injury. Chronic subdural hematomas (CSDH) occur 21 days after a head injury [4].

Subdural hematomas are regarded as a complex disease due to the formation of a membrane of capillaries surrounding the initial hematoma, which leads to recurrent small bleeding [5]. It is hypothesized that further hemostasis in the subdural space is prevented due to the fibrin degradation products from the original hematoma. The osmotic drive from the high protein content further elicits fluid into the hematoma space enlarging the initial hematoma [5].

The treatment of choice, in most cases, is a surgical intervention that allows for significant improvement of symptoms and brain decompression. Surgical intervention involves the patient undergoing general anesthesia with a burr hole craniostomy draining the liquified hematoma [6]. Iliescu delineated the current treatment of subdural hematomas requiring removal via surgical intervention. However, various surgical techniques demonstrated a potential relapse in bleeding [7].

It is hypothesized that hyperfibrinolysis can lead to the enlargement and liquefaction of CSDH. Therefore, pharmacologic interventions to restrict fibrinolysis have been implored to establish a less invasive approach as opposed to the current standard of treatment. Tranexamic acid (TXA) is a synthetic lysine-analog that has been identified as an alternative, non-invasive option to treat patients presenting with a subdural hematoma but who are poor surgical candidates [2]. TXA acts as an antifibrinolytic by inhibiting the binding of plasminogen to fibrin as well as inhibiting plasmin formation [8]. Current literature supports the efficacy of TXA versus surgical intervention, however, further research is warranted to justify the usage of TXA.

## Case presentation

**Figure 1:**
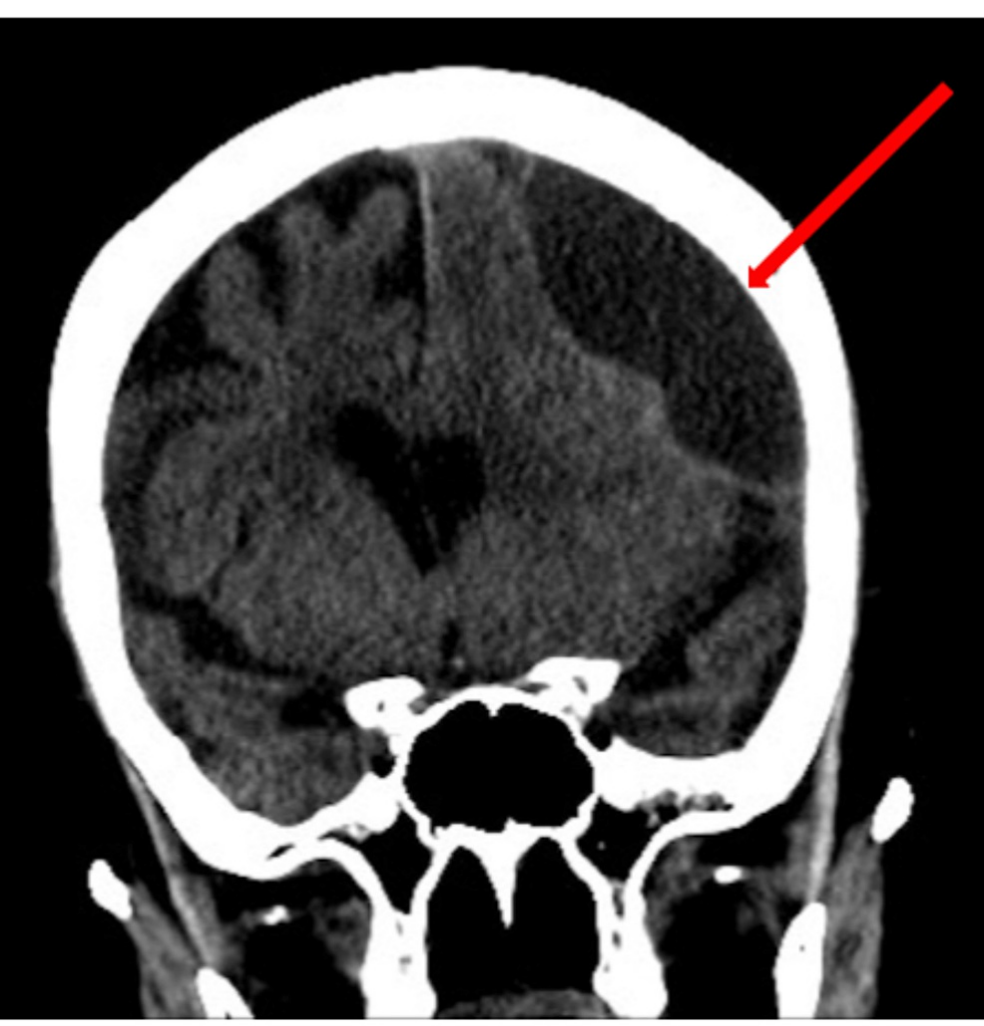
Initial CT scan displaying the 130 ml hematoma with concurrent 7 mm of midline shift

**Figure 2:**
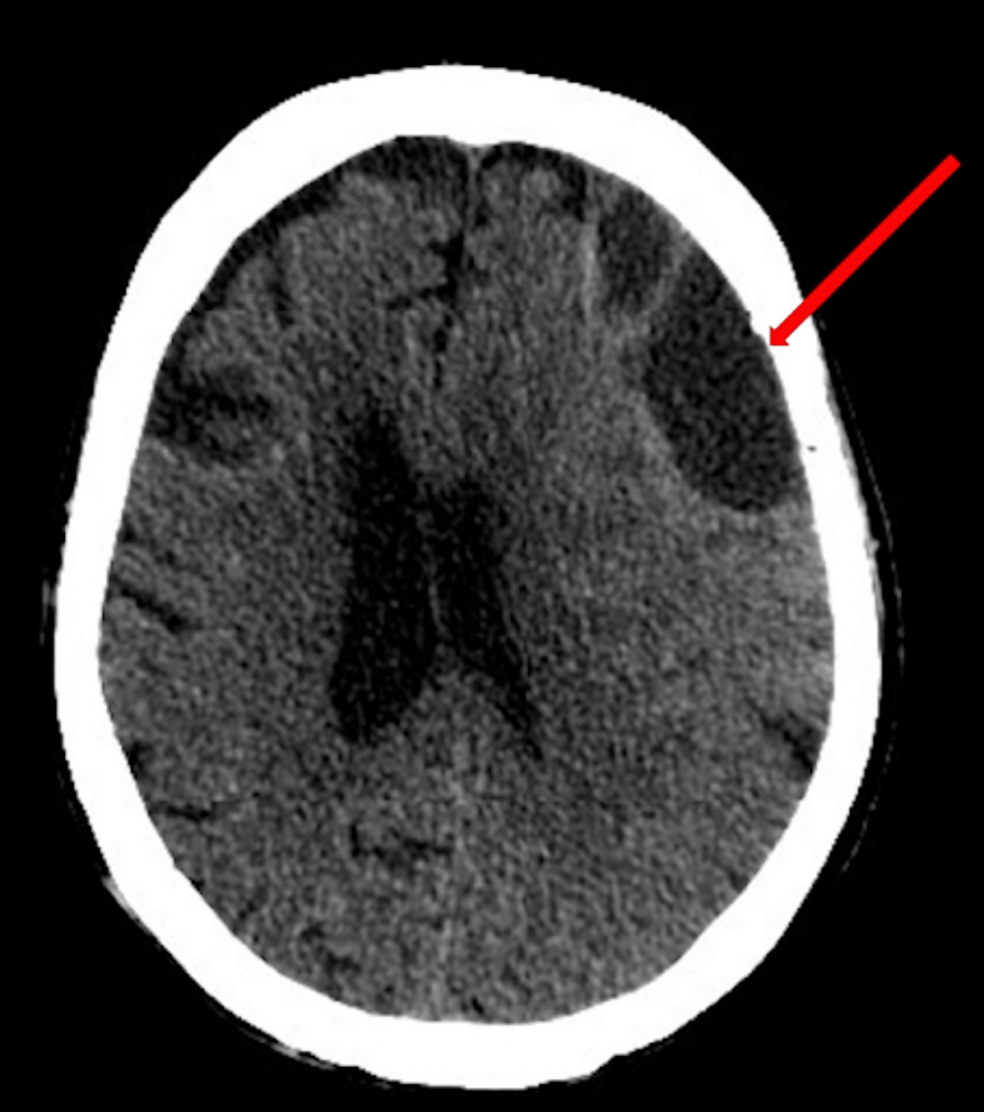
Initial CT scan displaying the 130 ml hematoma with concurrent 7 mm of midline shift

**Figure 3:**
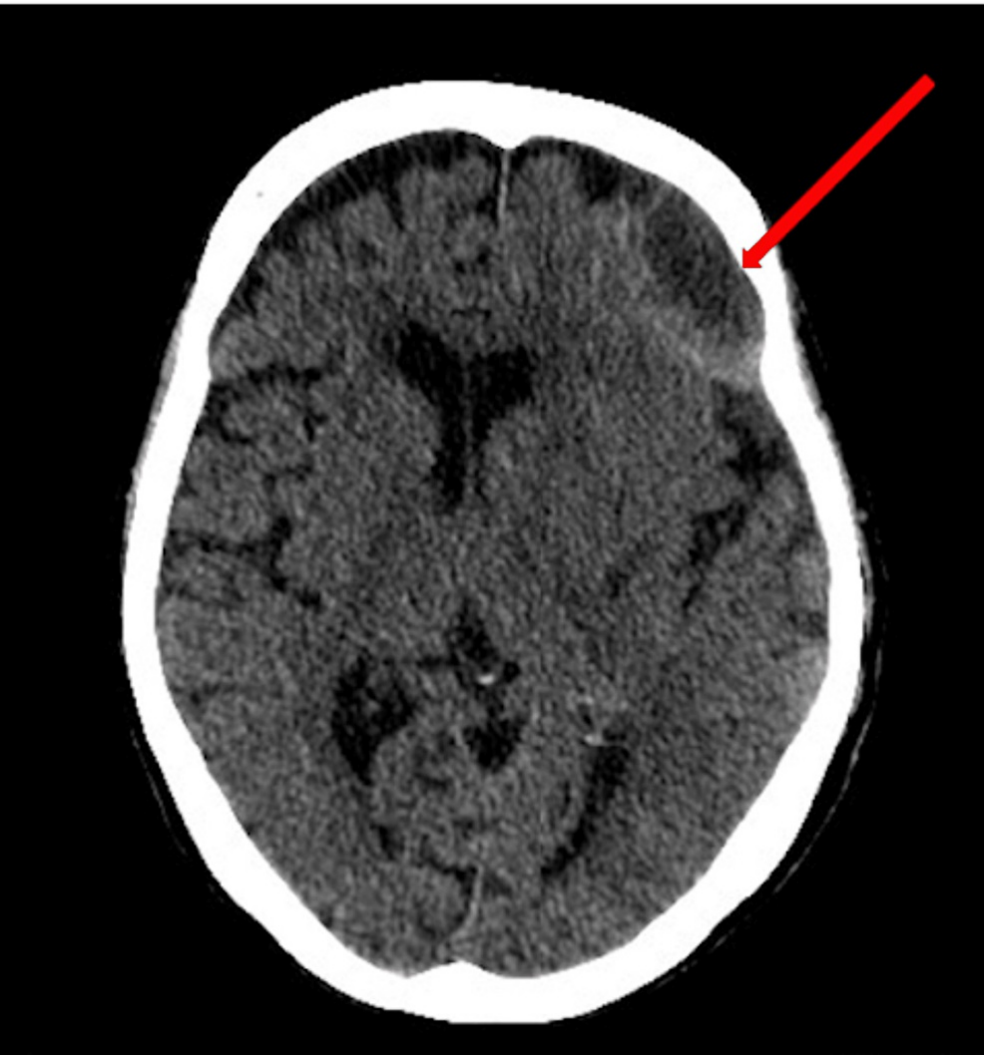
Initial CT scan displaying the 130 ml hematoma with concurrent 7 mm of midline shift

This case involved a 90-year-old female who presented with a code stroke with the chief complaint of right lower extremity weakness. The patient presented with a ground-level fall occurring two weeks ago along with a past medical history of uncomplicated hypertension, hyperlipidemia, and diabetes. On initial presentation, the patient's vitals were within normal limits and physical examination revealed pupils that were equal, round, reactive to light, and accommodating. Deep tendon reflexes were absent in the biceps, brachioradialis, knee, and ankle. Furthermore, the patient was unable to walk and was only alert to person and time. After a stroke series computed tomography (CT) panel, a left frontal multiloculated subdural hematoma was found measuring 130 mL with mass effect and a midline shift of 7 mm (Figures 1, 2, 3). 

Upon admission to the intensive care unit, the patient was recommended a craniotomy for hematoma evacuation or access to hospice for comfort care. A second opinion was sought after by the family which resulted in the administration of TXA. The patient was started on 650 mg of TXA (by mouth) twice daily for eight weeks.

**Figure 4:**
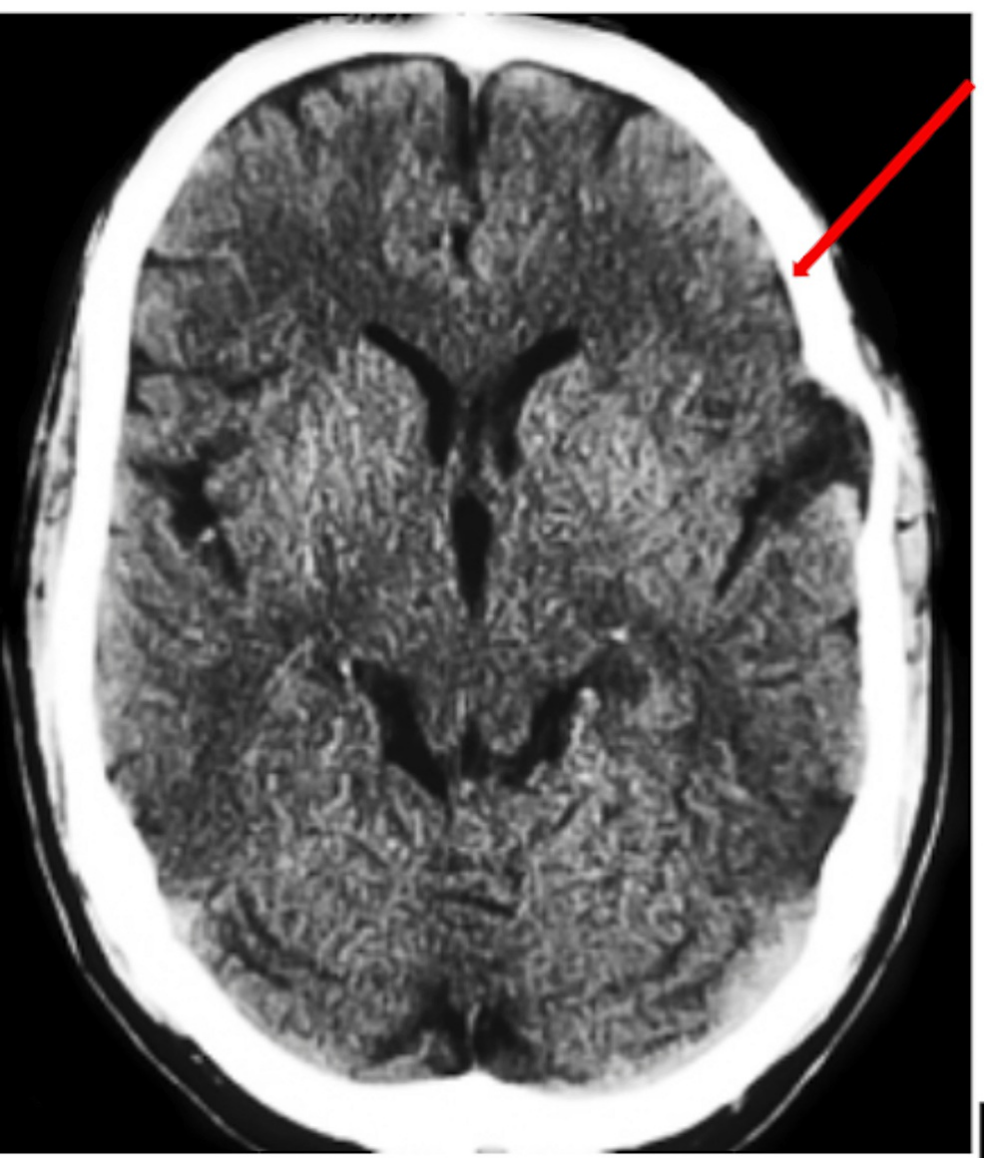
Final CT scan displaying final hematoma volume of 10 ml and less than 2 mm of midline shift

**Figure 5:**
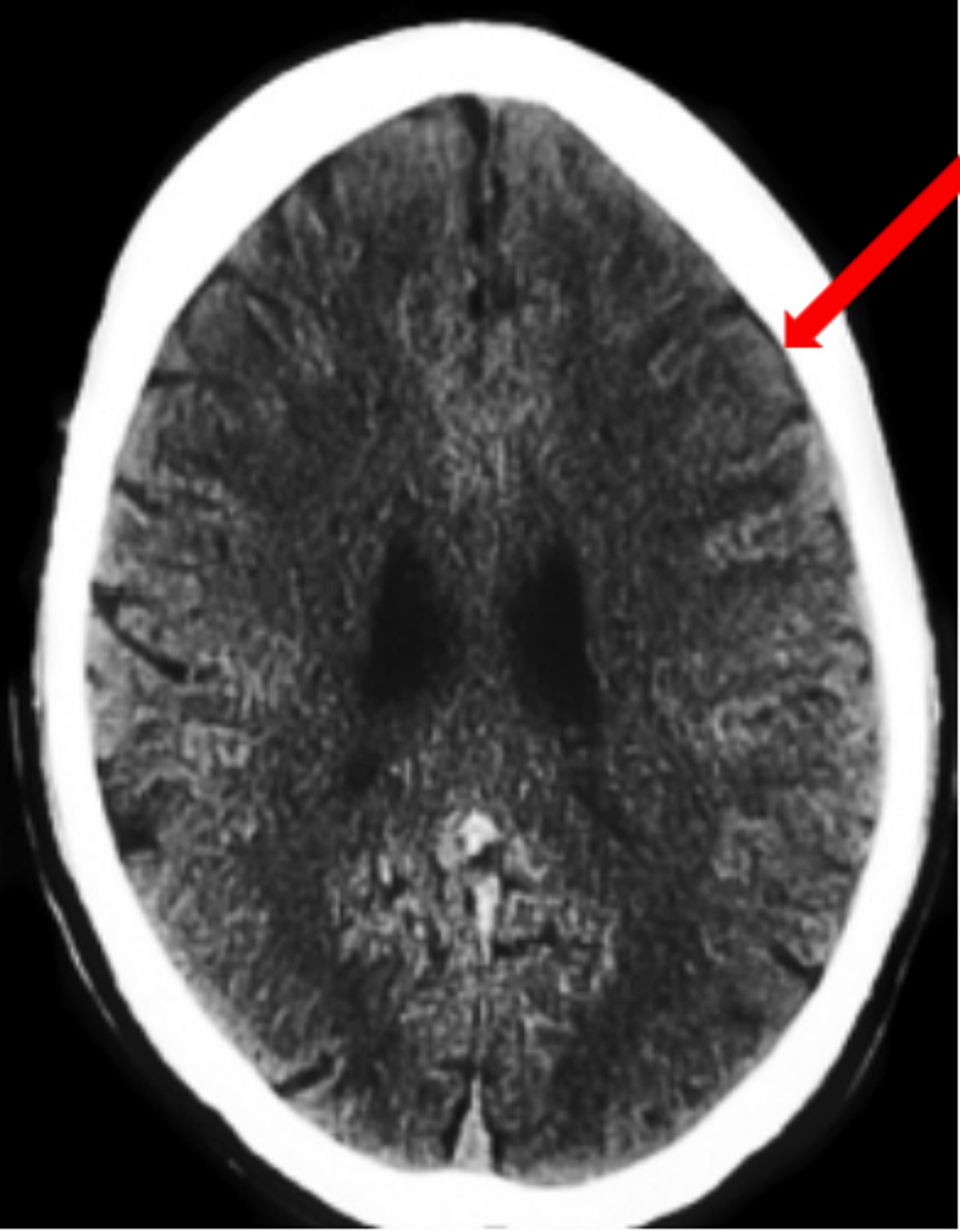
Final CT scan displaying final hematoma volume of 10 ml and less than 2 mm of midline shift

A CT ordered five weeks after the initiation of the TXA regimen illustrated hematoma resorption with a subsequent hematoma volume of 31 mL. Final hematoma measurements showed a final hematoma volume of 10 mL and a midline shift of less than 2 mm demonstrating significant reabsorption with an accompanying decrease of midline shift (Figures 4, 5). After the full completion of a TXA course, the patient achieved baseline mobility and was alert to person, place, and time.

## Discussion

We discuss a case of a 90-year-old woman who presented with a subdural hematoma treated with tranexamic acid. After treatment with TXA the patient was able to repossess baseline motor activity and baseline cognitive function and had no complaints of weakness. Subsequent CT has further validated the results by showing significant hematoma resorption with no adverse events occurring over the eight-week treatment regimen duration. The usage of TXA as a non-invasive treatment for subdural hematomas has demonstrated efficacy in the treatment of this patient and the implications of this treatment should be further implored.

The patient described in this study was prescribed a dosage regimen that was finalized at 650 mg, twice daily by mouth for eight weeks. This dosing regimen was established through the use of the Tranexamic Acid in Chronic Subdural Hematomas (TRACS) and Tranexamic Acid to Prevent OpeRation in Chronic Subdural Hematoma (TORCH) clinical trials that measured the efficacy of TXA in the treatment of chronic subdural hematomas [9,10]. In these trials, the dosages of TXA varied from 750 mg daily (TRACS) to 500 mg, twice daily (TORCH) [9,10]. In regards to the duration of treatment, the TRACS trial suggests the usage of TXA until hematoma resolution with concomitant CT imaging throughout the duration of treatment or an upper limit of 20 weeks of treatment is reached [9]. However, neither trial was able to establish a definitive standard dosage and duration of TXA administration.

According to the Brain Trauma Foundation, an SDH with a thickness >10 mm or midline shift >5 mm on CT should use surgical intervention irrespective of Glasgow Coma Scale (GCS) [11]. Furthermore, patients presenting with GCS <9 and SHD <10 mm and midline shift <5 mm must undergo surgical intervention if the GCS decreased by 2 or more points in a specific time range, physical exam changes, or with an intracranial pressure (ICP) of greater than 20 mmHg [11].

Surgical treatment for the evacuation of subdural hematomas varies depending on the preference of the neurosurgeon. The three main treatments described in current literature include burr hole craniostomy, single drill twist trephination, and craniotomy [4]. Although various surgical treatments are detailed, the most favored treatment is still at the neurosurgeon’s discretion.

Middle meningeal artery embolization is an adjuvant or alternative treatment that has been described recently for the treatment of chronic subdural hematomas. In a study conducted by Joyce et al., the researchers analyzed the usage of middle meningeal artery embolization in 121 elderly individuals [12]. Utilization of embolization techniques demonstrated significant reabsorption of hematomas and the researchers reported that embolization had stabilized or improved in 91% and 98% of the elderly (65yo - 79yo) and advanced elderly groups (>80 yo) [12]. The study also demonstrated surgical rescue being necessary in 4.6% and 7.8% of cases, and the overall mortality was 8.6% and 3.9% for elderly and advanced elderly patients [12]. The embolization technique is characterized as minimally invasive in nature but still requires a procedure to take place. This technique also further demonstrates a preferential shift towards non-invasive treatment for subdural hematomas and away from surgical intervention, the current standard of treatment.

The usage of TXA in the treatment of subdural hematomas has been described several times in current literature. In a study conducted by Kutty et al., 27 patients with subdural hematomas were treated with TXA [13]. They found 27 patients with subdural hematomas whose bleeds had a mean volume of 135.62 ± 92.90 mm and were mildly symptomatic upon presentation [13]. They found that after a 64.83 ± 24.8-day treatment of TXA the patient's bleeding showed resolution of the hematoma and none of the patient's hematomas progressed during the trial [13]. Another study conducted by Kageyama et al. describes a study of 21 patients who presented with subdural hematomas in which only three were treated previously with burr holes to evacuate the hematoma [6]. In the study, all patients were given TXA and showed marked reabsorption of hematoma with a decrease in mean volume from 58.5 ml to 3.7 ml. They also reported no hematoma recurrence or progression during the duration of the treatment. Both studies have shown the efficacy of TXA as a primary non-invasive treatment. In a case study conducted by Kutty et al., they evaluated the usage of TXA for conservative treatment for a subdural hematoma in a patient with thrombocytopenia stemming from concurrent HIV infection [14]. They reported the patient had resolution of the bleed on consecutive CT scans and upon follow up showed no deficits. They concluded that conservative treatment of subdural hematoma is effective in those identified as high risk for surgery [14]. For patients that are not suitable for surgical intervention and are at an increased risk of subdural hematomas, the treatment with TXA offers a conservative therapeutic alternative.

The current prospective TRACS study has accentuated the usage of off-label use of tranexamic acid to treat subdural hematomas [9]. In the TRACS study conducted by Iorio-Morin et al., they describe a double-blind, randomized, placebo-controlled prospective study analyzing the usage of TXA for subdural hematomas over the course of 20 weeks [9]. The TRACS study involves the enrollment of 130 patients to evaluate hematoma resorption. In addition, it is one of the first prospective studies to analyze the usage of TXA for the treatment of subdural hematomas [9]. The study on TRACS evaluates the growing usage of TXA as a subdural hematoma treatment and enables further exploration of TXA as a non-invasive treatment.

The current data on TXA for chronic subdural hematomas are limited with several studies in progress. Due to the limited information, TXA cannot be considered a standard treatment at this time, unlike other surgical interventions. The effectiveness of TXA as a treatment through this case description and current literature does advocate for further research into using TXA as a treatment for subdural hematomas.

## Conclusions

This case report demonstrates the effectiveness of TXA in patients suffering from CSDH. In this case report, pharmacological intervention with TXA administered to a 90-year-old patient with a CSDH demonstrated favorable outcomes including, diminished hematoma size accompanied by increased muscle strength. Similarly, current literature delineates the efficacy of TXA in patients with CSDH. Further studies are recommended to assess outcomes with the use of TXA in various patient populations. However, this case report continues to augment support for the use of TXA and encourage alterations in guidelines for patient populations that suffer from CSDH.
